# The Surgery of the Country Practitioner

**Published:** 1897-05

**Authors:** 


### THE SURGERY OF THE COUNTRY PRACTITIONER.

The surgical work of the country practitioner is varied in its scope, but it may be said that it consists to a very great extent in attention to emergencies. These may be of the most trivial kind, but they may also be of an appalling nature, such as try the souls of men, and it is often in this sort of work that the country doctor shows a power of correct and resolute decision which carries the day in the face of odds that appear to be insurmountable.

In surgery, as well as in medicine, the personal equation of the practitioner is a factor of paramount importance, and of all the elements which concur in the achievement of success, there is none of greater value than the possession of the rare faculty of so-called common-sense. There was a justly celebrated surgeon in this city whose terse description of the operation for strangulated hernia is well worth remembering. He said, "Cut through the Latin names and get to the gut." It takes but a moderate degree of skill to perform many an important operation, and a clear understanding of what is to be accomplished, with the utmost care never to cut unless you are sure of what your incision is going to accomplish, will often bring about a good result which no amount of theoretical knowledge alone could have achieved. In the treatment of fractures and dislocations the man with a mechanical bent of mind can accomplish wonders. In the treatment of wounds accurate coaptation of severed surfaces and a thorough understanding of cleanliness are the most important points. It is evident, that in surgery, an immense amount can be done by the man of quick perception and ready understanding and we are thankful to be able to say that it is our belief that such individuals are many and widely scattered over the land, helpful, energetic, with faculties keen and ready to bear upon the most knotty problems that may be presented by the appalling emergencies which so often suddenly arise in the practice of our country doctors.—*International Journal of Surgery.*

